# Assessment of in situ nest decay rate for chimpanzees (*Pan troglodytes ellioti* Matschie, 1914) in Mbam-Djerem National Park, Cameroon: implications for long-term monitoring

**DOI:** 10.1007/s10329-019-00768-3

**Published:** 2019-10-28

**Authors:** Serge Alexis Kamgang, Tuneu Corral Carme, Kadiri Serge Bobo, Ekwoge Enang Abwe, Mary Katherine Gonder, Brice Sinsin

**Affiliations:** 1 Garoua Wildlife School, Face aéroport International de Garoua, PO Box 271, Garoua, Cameroon; 2 Ministry of Forestry and Wildlife, Yaoundé, Cameroun; 3 Department of Animal Biology, Plant Biology and Ecology, Autonomous University of Barcelona, Bellaterra, 08193 Barcelona, Spain; 4 Department of Forestry, Faculty of Agronomy and Agricultural Sciences, University of Dschang, PO Box 222, Dschang, Cameroon; 5 Department of Biology, Drexel University, Philadelphia, PA 19104 USA; 6 Laboratory of Applied Ecology, Faculty of Agricultural Science, University of Abomey-Calavi, 01, PO Box 526, Cotonou, Bénin

**Keywords:** Mbam-Djerem National Park, Local nest decay rate, Chimpanzee density, Indirect survey, Conservation strategies

## Abstract

Accurate assessment of great ape populations is a prerequisite for conservation planning. Indirect survey methods using nest and dung, and a set of conversion parameters related to nest decay rates, are increasingly used. Most surveys use the standing crop nest count (SCNC) method, whereby nests are counted along transects and the estimated nest density is converted into chimpanzee density using an often non-local nest decay rate. The use of non-local decay rate is thought to introduce substantial bias to ape population estimates given that nest decay rates vary with location, season, rainfall, nest shape, and tree species used. SCNC method has previously been applied in Mbam-Djerem National Park (MDNP) in Cameroon, for chimpanzee surveys using a non-local nest decay rate. This current study aimed to measure a local nest decay rate for MDNP and implications for chimpanzee population estimates in the MDNP. The mean nest decay rate estimated using a logistic regression analysis was 127 [95% CI (100–160)] days. Moreover, the results suggested that rainfall strongly influenced the nest decay rate over the early stage of the lifetime of the nests. The study confirms that estimates of chimpanzee density and abundance using non-local decay rates should be treated with caution. Our research emphasized the importance of using local nest decay rates and other survey methods which do not depend on decay rates to obtain more accurate estimates of chimpanzee densities in order to inform conservation strategies of these great apes in MDNP.

**Electronic supplementary material:**

The online version of this article (10.1007/s10329-019-00768-3) contains supplementary material, which is available to authorized users.

## Introduction

The Congo Basin rainforest is the second largest wilderness area on the planet after the Amazon (De Wasseige et al. 2012), representing 70% of the African forest cover (Guinness 2010) and sheltering most populations of African great apes (Fruth and Hohmann 1996; Williamson et al. 2013). This forest and the fauna inhabiting it are threatened by habitat loss, fragmentation, bushmeat hunting, climate change, emerging infectious diseases, and other activities such as logging and mining (Huijbregts et al. 2003; Walsh et al. 2003; Bermejo et al. 2006; Hart et al. 2008; Spehar et al. 2010; Wevers et al. 2011; IUCN 2014; Katsis et al. 2018; Hicks et al. 2010), which are exacerbated by poverty and pervasive corruption (Walsh et al. 2003; Hart et al. 2008). As a consequence of all these factors, great ape populations have experienced a precipitous decline within the last 40 years (Fruth and Hohmann 1996; Walsh et al. 2003; Williamson et al. 2013). Thus, large-scale conservation measures are needed urgently in order to avoid further decline of great ape populations as well as to mitigate the various threats (Spehar et al. 2010; Cameron et al. 2016).

The common chimpanzee (*Pan troglodytes,* Blumenbach 1799) has the largest geographic distribution of all nonhuman great ape species and is located discontinuously from southern Senegal across the forested belt north of the Congo River to western Tanzania and western Uganda (Williamson et al. 2013; IUCN 2014; Humle et al. 2016). Four subspecies are recognized: the western chimpanzee (*P. t. verus*, Schwarz 1934), the Nigeria-Cameroon chimpanzee (*P. t. ellioti*, Matschie 1914), the central chimpanzee (*P. t. troglodytes*, Blumenbach 1799), and the eastern chimpanzee (*P. t. schweinfurthii*, Giglioli 1872) (Gonder et al. 2006; Gonder et al. 2009; Prado-Martinez et al. 2013). With less than 9000 wild individuals remaining, *P. t. ellioti*, the focal subspecies of this study, has the smallest geographic range and the smallest population size of all chimpanzee subspecies (Gonder et al. 1997; Caldecott and Miles 2005; Morgan et al. 2011). All four chimpanzee subspecies are classified as Endangered by the IUCN Red List of threatened species (Oates et al. 2016). Given its more limited geographical distribution and numbers, *P. t. ellioti* merits more urgent conservation action (Morgan et al. 2011).

An effective management of chimpanzees requires an accurate understanding of their population status including density, abundance, distribution, threats, and population trends in order to prioritize strategies needed for their conservation (Kühl 2008; Kalan et al. 2016; Morgan et al. 2011). Chimpanzees are elusive and most populations are assessed indirectly using nest counts (Plumptre 2000; Laing et al. 2003; Kühl 2008; Spehar et al. 2010; Head et al. 2013; Hicks et al. 2014; Howe et al. 2017; Kamgang et al. 2018). In all great ape species, each weaned individual builds each evening a nest made of leaves, branches, and other vegetal material in which to spend the night (Tutin and Fernandez 1984). Nests provide comfort, thermoregulation, and protection against predators and parasites (Kingdon and Largen 1997; Anderson 2000; Pruetz et al. 2008; Stewart et al. 2011; Koops et al. 2012; Samson and Hunt 2012). These nests can remain intact from a few days to several months (Tutin and Fernandez 1984; Mathewson et al. 2008) and have thus been used to ascertain the presence and estimate the population of chimpanzees.

Over the past decades; circumstantial evidence (usually animal dung and nests) has been increasingly used in wildlife surveys (Krebs 1989; Vernes 1999; Laing et al. 2003; Walsh and White 2005; Nzooh et al. 2015; Nzooh et al. 2015). Nest or dung surveys have been used to estimate abundance for several mammal species such as bonobos *Pan paniscus* (Serckx et al. 2014), western lowland gorillas *Gorilla gorilla* (Walsh and White 2005; Haurez et al. 2014; Tsakem et al. 2015), Bornean orangutans *Pongo* *pygmaeus* (Mathewson et al. 2008; Spehar et al. 2010), African elephants *Loxodonta cyclotis* (Fay 1991; Barnes et al. 1997; Nchanji and Plumptre 2001; Barnes and Dunn 2002; Jathanna et al. 2015), European deer *Capreolus capreolus,* and kangaroos *Macropus rufus* (Vernes 1999).

The standing crop nest count (SCNC) method is typically used to estimate chimpanzee densities from nest counts along transects. In this method, nests are surveyed once and nest density is converted to population density estimates using a production rate and a nest decay rate (Tutin and Fernandez 1984; Morgan et al. 2006). The nest creation rate can be obtained by direct monitoring of habituated chimpanzees (Tutin and Fernandez 1984), while estimating nest decay rate in unhabituated populations requires more time and resources, and involves monitoring a sufficient number of fresh nests from the time they are built to the time they disappear (Plumptre and Reynolds 1996). Most surveys use a non-locally-acquired nest decay and production rate, although these two parameters are not reliably comparable across sites (Laing et al. 2003; Buij et al. 2003; Kühl 2008), as nest decay rates might be affected by a number of local factors such as climatic conditions, habitat type, and plant species used to build the nests (Laing et al. 2003; Maisels et al. 2009; Singleton 2009; Stokes et al. 2010). The SCNC method to estimate great ape densities is financially affordable for most protected areas, but few studies on nest production and decay rates have been conducted (Plumptre and Reynolds 1996; Morgan et al. 2006).

In comparison, the mark nest count method does not use the nest decay rate but it takes into account the length of time between survey intervals (Mathewson et al. 2008; Spehar et al. 2010; Ndimbe et al. 2013). All nests are counted and marked during the first survey and on subsequent surveys only new nests are counted and marked. However, the time interval between two consecutive surveys should be shorter than the minimum time for the nest to disappear (Hashimoto 1995; Plumptre and Reynolds 1996). Several surveys have been carried out in the MDNP to estimate chimpanzee abundance (Maisels et al. 2000, 2009; Kamgang et al. 2018). However, all of these previous surveys used the SCNC method with a non-locally acquired nest decay rate, which may not give a robust estimate of chimpanzee densities in the MDNP.

Therefore, our study aimed to assess the local nest decay rate of chimpanzees in the core area of the MDNP, as well as to detect some of the environmental factors influencing nest decay rates. As no assessment of nest decay rates has been conducted previously in this area, our research was essential to achieve more precise estimates of *P. t. ellioti* density and to shape effective management practices for long-term monitoring in the MDNP.

## Methods

### Study area

**Fig. 1:**
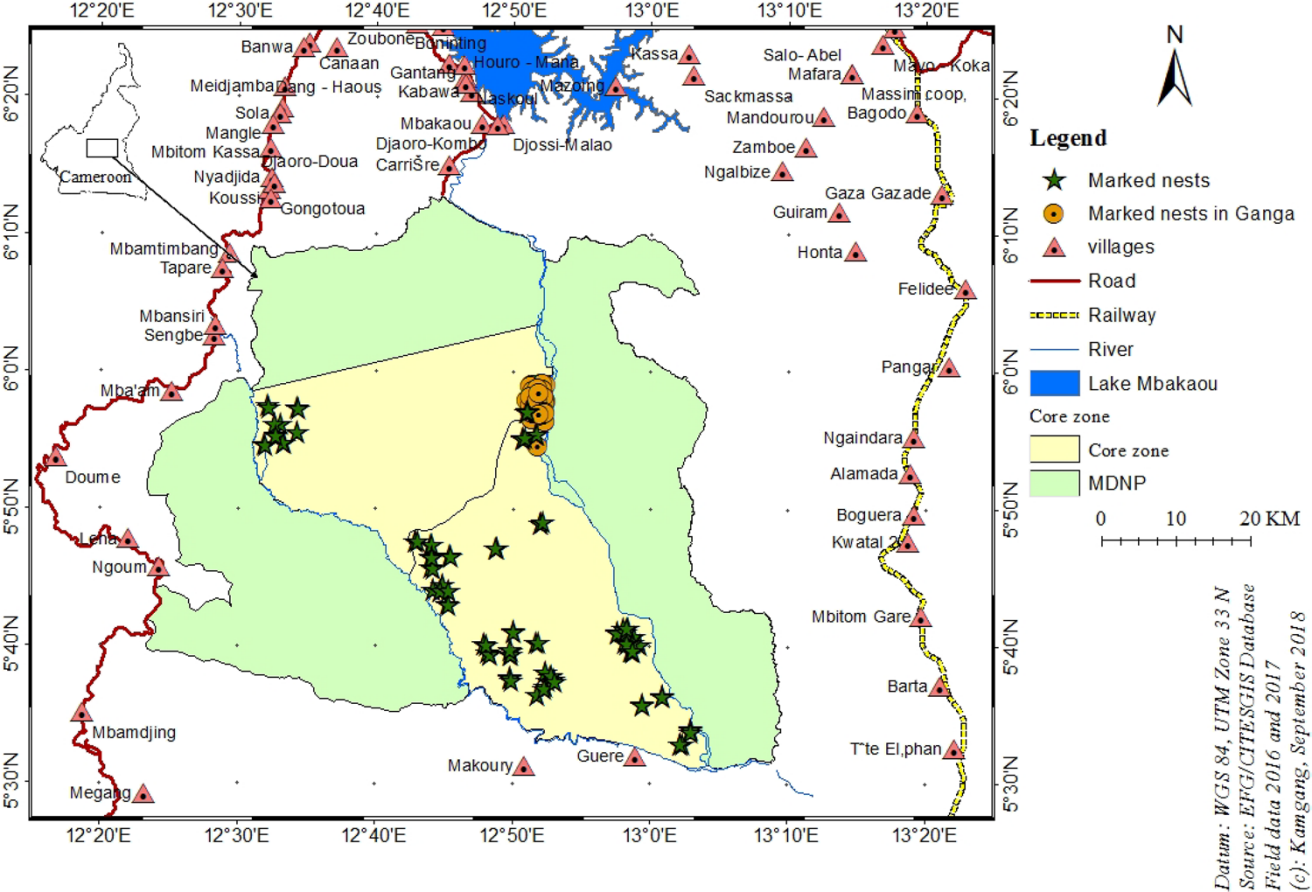
The Mbam-Djerem National Park showing the surrounding villages and the locations where chimpanzee nests were marked in the core zone

The forest–savannah transition zone in central Cameroon is of high importance as it preserves interconnecting corridors between populations of the two chimpanzee subspecies (*P. t. ellioti* and *P. t. troglodytes*), which gives the region a unique importance (Mitchell et al. 2015). This region includes the MDNP (5° 72′ N, 12° 68′ E), which extends 4165 km^2^, with a core zone of 1662 km^2^ and is the largest protected area of forest–savannah mosaic habitat in Cameroon (Bobo et al. 2006; WCS 2017). About half of the park in the southern sector is covered by lowland tropical forest and the other half, in the northern sector, is covered by Sudano-Guinean tree and woodland savannah with a wide forest–savannah mosaic in between, which promotes a very high level of biodiversity (White 1983; WCS 2017). The climate in this region has two seasons: a rainy season from mid-April to mid-October, and a dry season from mid-October to mid-April (MINFOF 2007; Abwe et al. 2019). The relief is nearly flat, with a north-to-south altitudinal decline from 930 to 650 m asl (Bobo and Weladji 2011). The park is drained by the Djerem River, which serves as a permanent water source for wildlife and more than 74 human communities around the park. Human activities such as poaching, illegal logging, and illegal grazing are among the main threats to the wildlife in the MDNP landscape (Bobo and Weladji 2011; WCS 2017). The study site was chosen based on information previously gathered on chimpanzee nest abundance and distribution in the core zone of the MDNP (Maisels et al. 2000, 2009; Kamgang et al. 2018) (Fig. 1).

### Data collection

Nest decay rate can be calculated by either the prospective or the retrospective method (Laing et al. 2003; Stokes et al. 2010; Dutton 2012). The prospective method consists of marking fresh nests at a given time and revisiting them at regular intervals until they disappear (Mathewson et al. 2008). This method may lead to bias if seasonal fluctuations exist in nest decay rates (Laing et al. 2003). In contrast, the retrospective method, which we used in the current study, consists of marking fresh nests at different periods of time and revisiting them only once in order to verify whether or not they have decayed. It is expected that during the revisit, the number of nests remaining from the first visit will be fewer than those marked later. Using this technique, a period of time for a visit is preselected and the mean decay rate of the nests already present can be estimated (Laing et al. 2003; Mathewson et al. 2008).

### Nest marking

We carried out 2-week surveys monthly between January 2016 and March 2017. During this period, we patrolled areas of high activity for chimpanzee in the core zone to locate and mark fresh chimpanzee nests (less than 24 h old). All nests were marked with codes referring to the survey number (S1, S2,…, S6), the nest group (G1, G2,…, Gn) and the number of marked nests of the same group (N1, N2,…, Nn). We also marked other fresh nests as we walked transects during biomonitoring activities in 2016 (Kamgang et al. 2018). For each nest marked nest and nesting site, we recoded the following information: (1) date, (2) vegetation type (gallery forest, colonizing forest or savannah), (3) geographic coordinates, (4) topography of the site (flat, gentle or steep), and (5) total rainfall during the first month of the nest’s lifetime.

### Rainfall data

**Fig. 2:**
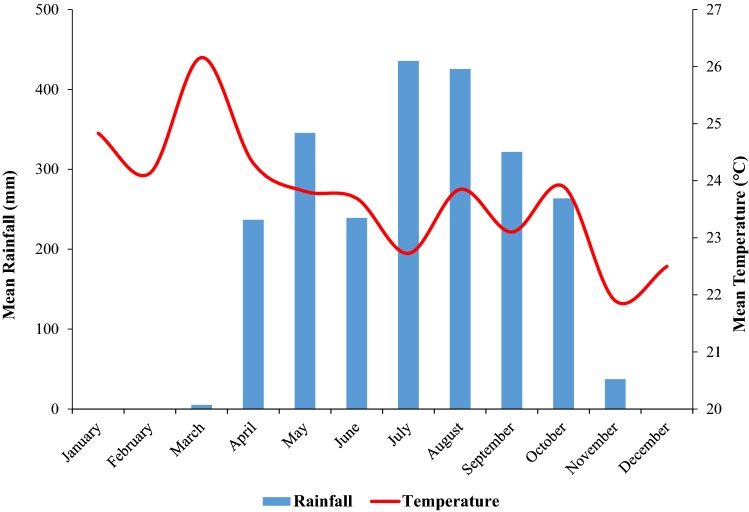
Climate graph of the MDNP over the period of the study (2017)

Rainfall data over the same time period as the study were obtained from the Wildlife Conservation Society office at Mbakaou, at the northern edge of the MDNP (Abwe et al. 2019). For each nest marked, we considered the rainfall of the same month the nest was built. We then plotted the climate graph showing rainfall variation in 2017 (Fig. 2).

### Revisiting marked nests

We revisited all marked nests once (Supplementary File 1) to attest whether or not they were still present. We marked nests that were present as (1) and those that were absent as (0) (Supplementary File 2). We considered a nest absent when we were no longer able to identify its structure, that is, the leaves had all decayed and only the framework remained visible (Van Schaik et al. 1995; Plumptre and Reynolds 1996; Buij et al. 2003) (Supplementary File 3).

### Data analysis

We used the software programme QGIS 3.2 Bonn to map the geographic location of all marked nests in the study area (Fig. 1). We conducted a logistic regression in R to estimate the mean nest decay rate (Laing et al. 2003; Mathewson et al. 2008; Nzooh et al. 2015). We considered only two variables in this analysis: the predictor variable “age” and the response variable “absence/presence”. This model allowed us to plot the logistic regression curves of the nest age and to determine the number of days needed for 50% of the nests to be considered decayed (Eq. 1).

1 $$P\left( {Y = \frac{1}{{X_{i} }}} \right) = \frac{{e^{{\alpha + \beta X + \alpha_{i} }} }}{{1 + e^{{\alpha + \beta X + \alpha_{i} }} }},$$

where *α* is a constant and *β* the coefficient of time, and *X* representing the time (days).

We tested three logistic regression models in order to reduce the residual deviance following transformation of the predictor variable: the first model (Model 1) used non-transformed data, the second model (Model 2) used the negative-inverse transformation and the third model (Model 3) considered the negative-reciprocal-root transformation. We did not use the logarithmic transformation in this analysis, since sometimes it generates problems when fitting the upper tail of the logistic regression curve, which may result in a biased estimation of the mean nest decay rate (Laing et al. 2003). We tested the suitability of the models by comparing the Akaike’s Information Criterion (AIC) values and the residual deviance. Furthermore, we tested the model accuracy by assessing the area under the curve (AUC). We derived the mean nest decay rate and the confidence interval for each model by isolating the *X* factor from Eq. (1) using the coefficients α and β from each logistic regression (Eq. 2).

2 $$X = \frac{{\log \left( {\frac{1}{Y} - 1} \right) + \alpha }}{ - \beta }.$$

We also assessed whether or not the other recorded variables influenced the nest decay time. Therefore, we added “precipitation”, “topography”, and their interactions to each of the models tested previously in a second logistic regression analysis. In each case, we fitted a null model first and we added the other variables sequentially. We did not consider “habitat type” or “altitude” in the models since a high proportion of nests were found in forest and the observed altitude range was minimal (665–854 m asl). We assumed that with the present sample size, the effect of altitude on the mean nest decay rate would not likely be accurately detectable. We performed a third logistic regression analysis considering only “age” and “precipitation” as predictor variables.

Finally, we converted the nest density calculated in the previous study (Kamgang et al. 2018) to chimpanzee density and abundance by using the local nest decay rate obtained in each model. As in Kamgang et al. (2018), the nest production rate was one nest/day/weaned individual and the proportion of nest builders was one as well. We assessed chimpanzee density using Distance 7.2 software (Buckland et al. 2001; Thomas et al. 2010). For this density assessment, we used data from the 2016 survey as well as the local nest decay rate obtained from the current study. We then compared our results to those found by Kamgang et al. (2018) to show how both the chimpanzee density and abundance varied when different non-local and locally acquired nest decay rates were used, emphasizing the importance of the present study.

## Results

We marked and revisited a total of 309 fresh nests (Table 1) during this study. Among the 296 fresh nests marked in the Ganga region, we were only able to revisit 198 due to time constraints and nest accessibility. We also marked and revisited 111 nests from other high-activity chimpanzee areas to assess the effects of environmental variables on the nest decay rates.

**Table 1:** State of nests revisited (3 months after nest marking) in the Ganga region and other high-activity chimpanzee areas (HAA)

Sectors	Number of nests revisited	Number of nests absent	Number of nests present
Ganga region	198	166	32
Other HAA	111	46	65
Total	309	212	97

**Fig. 3:**
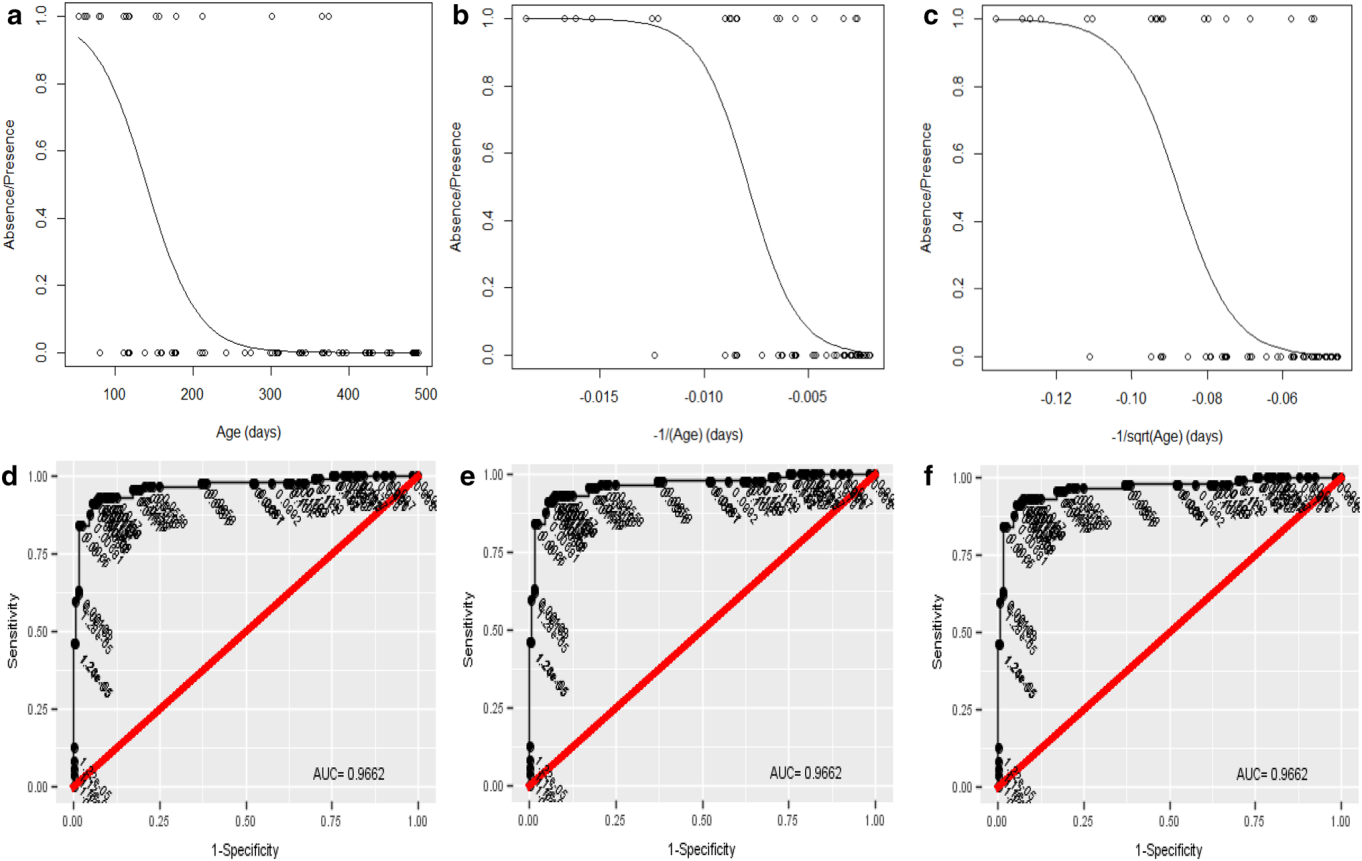
The effects of nest age on the probability of nest decay. At the top, the logistic regression curves fitted under the three models: **a** Model 1, **b** Model 2, and **c** Model 3. The* dots* show the recorded observations, being 1 for present nests and 0 for decayed nests. At the bottom, the ROC curves (**d**–**f**) under the respective model they belong to, and their corresponding AUC value

The results obtained in the first logistic regression analysis showed a clear correlation between “absence/presence” and “age” in the three models (*P* < 0.0001), with the probability of nests still being present decreasing significantly as age increased (Table 2, Fig. 3). The nest decay times (µ_X_) calculated were 141 [95% CI (109–186)] days in Model 1, 127 [95% CI (100–160)] days in Model 2 and 130 [95% CI (81–207)] days in Model 3 (Table 3). With a wider confidence interval range, Model 3 was less defined compared to Models 1 and 2. However, the three mean decay times obtained were similar. The same AUC was obtained in all Models (0.962) and the AIC was 175.29 in Model 1, 127.25 in Model 2, and 130.57 in Model 3. With the lowest AIC, we considered Model 2 the best model fitting the logistic regression curve. This model also showed the most reduced residual deviance (123.25) with the negative-inverse-transformation, followed by Model 3 with the negative-reciprocal-root-transformation (126.57), and finally Model 1 with no transformation of data (153.29).

**Table 2:** Results obtained from fitting the three logistic regression models considering “absence/presence” as the response variable and “age” as the predictor variable

Models		Residual deviance	Deviance change	df	df change	*P*	AIC*	AUC**
Model 1								
Null		367.35		308			175.29	0.962
+ Age		153.29	214.06	307	1	< 0.0001		
Model 2								
Null		367.35		308			127.25	0.962
+ (−1/age)		123.25	244.06	307	1	< 0.0001		
Model 3								
Null		367.35		308			130.57	0.962
+ (1/√age)		126.57	240.78	307	1	< 0.0001		

*Akaike information criterion

**Area under curve

**Table 3:** Estimated mean nest decay time (µ_X_) with the 95% confidence interval (CI) for each model

Models	µ_*X*_ (days)	95% CI
Model 1	141	[109–185]
Model 2	127	[100–160]
Model 3	130	[81–207]

In Model 1, *X* is non-transformed while in Model 2, *X* is negative-inverse transformed and in Model 3, *X* is negative-reciprocal root-transformed

**Fig. 4:**
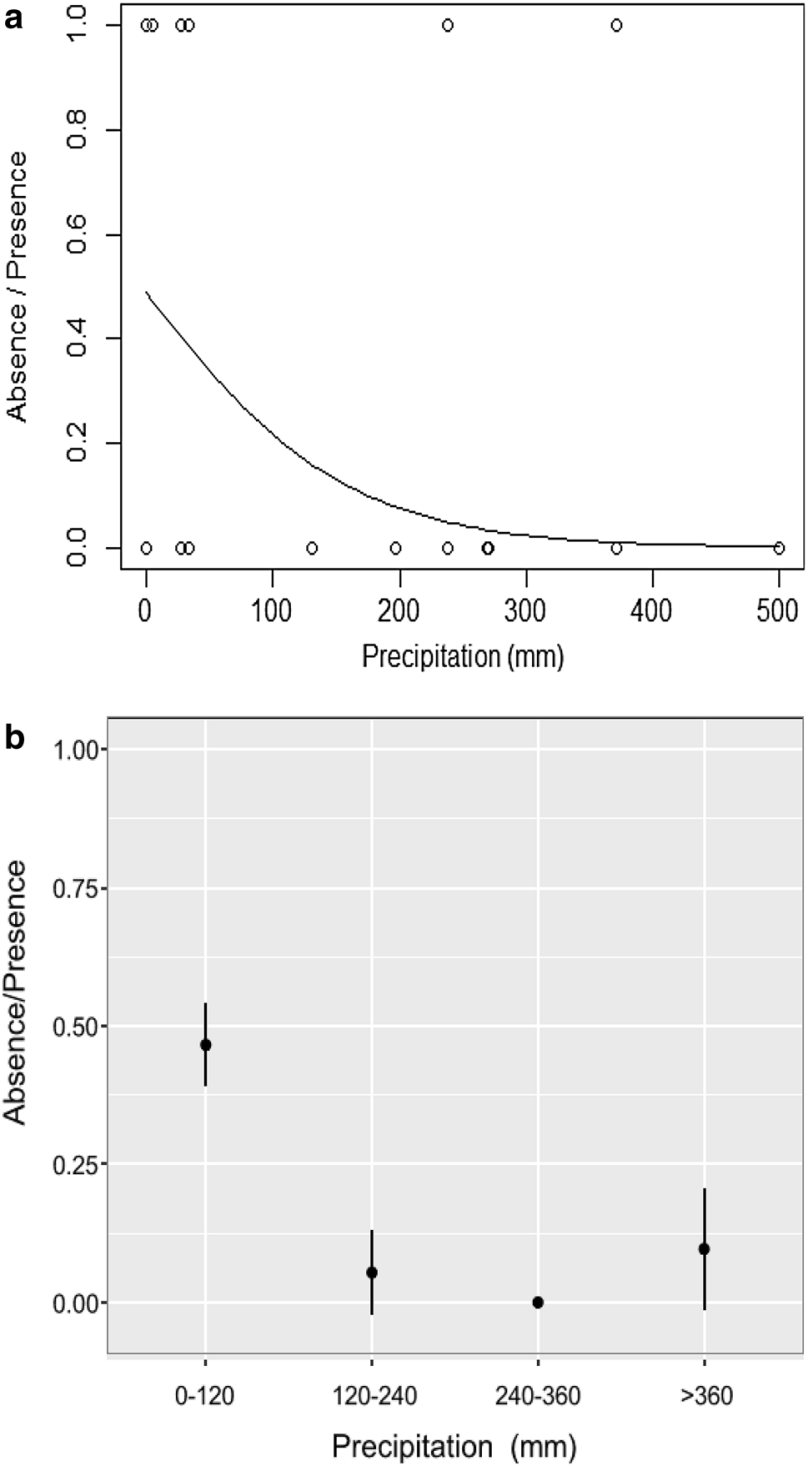
**a** The effects of “precipitation” on the probability of nest decay (logistic regression curve of “precipitation” fitted to nest data). **b** The effects of “precipitation” on the probability of nest decay (“absence/presence” mean value and its 95% confidence interval considering four ranges of the variable “precipitation” corresponding to the total rainfall during the first month of the nests: 0–120 mm, 120–240 mm, 240–360 mm, and > 360 mm)

**Fig. 5:**
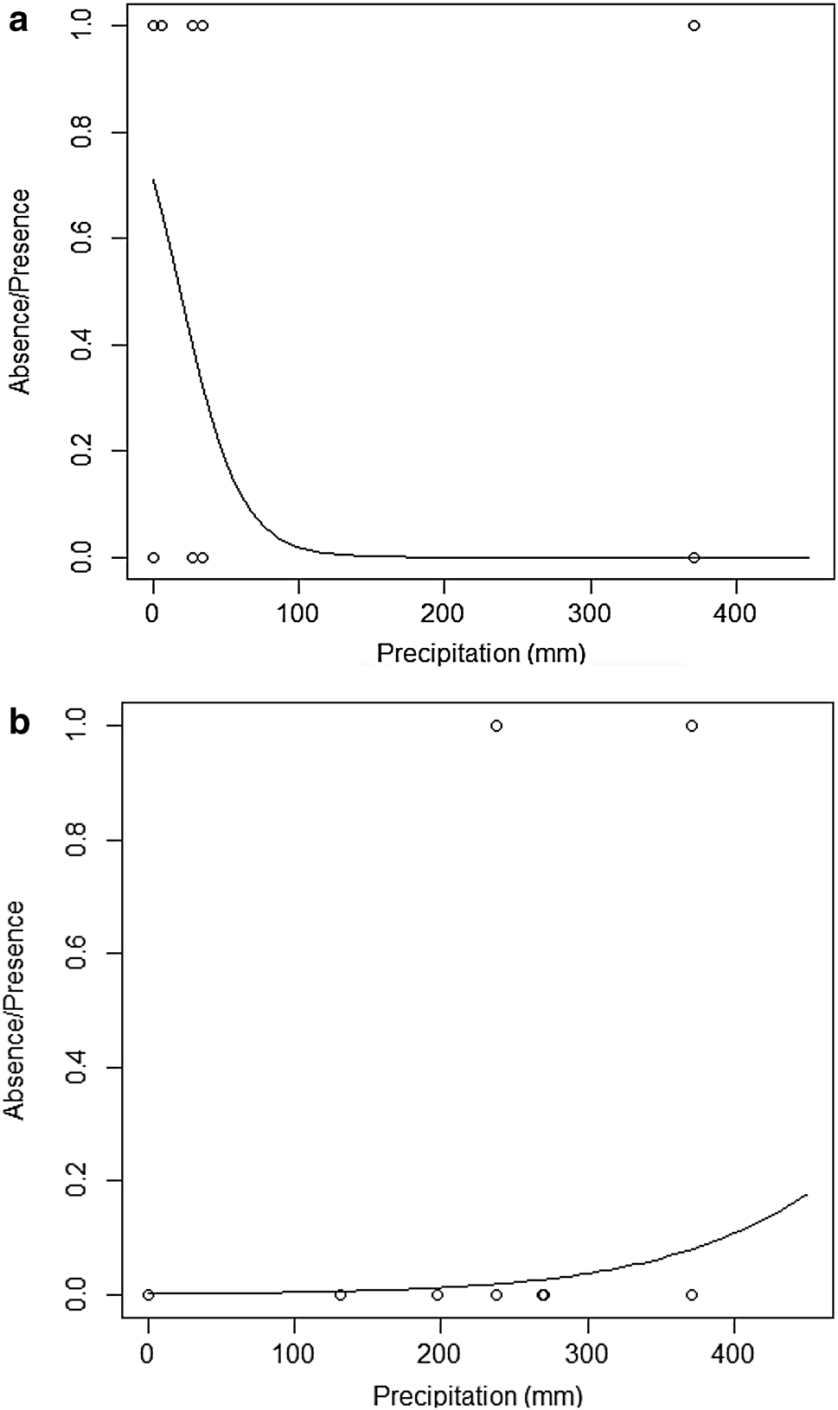
**a** The effects of the interaction “Age*Precipitation” (logistic regression curve of “Precipitation” fitted to nest data considering a nest age found between 0 and 250 days). **b** The effects of the interaction “age*precipitation” (logistic regression curve of “precipitation” fitted to nest data considering a nest age above 250 days)

In the second logistic regression analysis, the effects of “topography” and its interactions with other variables were not significant (*P* > 0.05) and hence, they were not considered in the final model (Supplementary File 4). The results obtained from the third logistic regression analysis without “topography” for Model 2 are presented in Table 4. With regards to the first logistic regression analysis, the variable “age” showed a strong effect on the decaying probability of nests (*P* < 0.0001). The effect of “precipitation” was also significant (*P* = 0.034), increasing the probability of nest decay as the total rainfall in the first month increases (Fig. 4a). Under low monthly rainfall (0–120 mm) the mean value of “absence/presence” was 0.47, which decreased below 0.2 when the monthly rainfall was higher (> 120 mm) (Fig. 4b). However, the interaction “age*precipitation” was not significant (*P* > 0.05). The nest age also influenced the probability of nest decay, especially during the first month of the nest (Fig. 5a, b). (Supplementary File 5).

**Table 4:** Results obtained from fitting the logistic regression model considering “absence/presence” as the response variable and “age” and “precipitation” as predictor variables

Model 2		Residual deviance	Deviance change	df	df change	*P*	AIC	AUC
Negative-inverse transformed								
Null		367.35		308				
		123.25	244.10					
+ (− 1/Age)				307	1	< 0.0001	124.77	0.966
					1			
						0.034		
			4.48					
				306				
+ Precipitation		118.77

We assessed the chimpanzee density using a nest decay rate of 127 days from the most suitable model (Model 2), and obtained a value of 0.59 [95% CI (0.41–0.86)] chimpanzees/km^2^ with an abundance of 987 [95% CI (683–1427) weaned chimpanzees. We then compared these results to those obtained from the 2016 survey (Table 5).

**Table 5:** Chimpanzee density and abundance at the core area of MDNP and their respective 95% confidence interval (CI) for each mean decay time

Nest decay time (days)	Chimpanzees/km^2^ [CI]	Chimpanzee abundance [CI]	Studies
88	0.83 [0.32–2.11]	1396 [535–3643]	(Kamgang et al. 2018)
120	0.61 [0.23–1.59]	1026 [397–2650]	
221	0.33 [0.12–0.86]	557 [216–1439]	
127	0.59 [0.41–0.86]	987 [683–1427]	Present study
130	0.58 [0.40–0.84]	964 [667–1394]	
141	0.53 [0.37–0.77]	889 [615–1285]	

## Discussion

Techniques for estimating the abundance and population status of chimpanzee using nests are well known. Most studies still rely on non-locally derived nest production and decay rates, making the derived great ape densities questionable. In this study, we assessed the nest decay rate for chimpanzees in MDNP and tested the influence of some factors on this parameter. The obtained value allowed us to estimate with greater accuracy the density of chimpanzees in the MDNP, which could to be useful for the long-term monitoring of populations across the park and adjacent areas.

The calculations of the probability of decay of chimpanzee nests is mainly based on nest age, although other factors such as precipitation, topography, and plant material could be important (Laing et al. 2003; Walsh and White 2005; Mathewson et al. 2008). Our mean nest decay rates were similar to those found in Campo Ma’an National Park (south-western Cameroon, 420 km from MDNP), estimated at 130 days (Matthews and Matthews 2004), as well as in Lopé National Park (Gabon, 710 km from MDNP), estimated at 114 days (Tutin and Fernandez 1984). In contrast, very different decay rates have been found at other sites such as 46 days at Budongo Forest (Uganda, 2150 km from MDNP) (Plumptre and Reynolds 1996), 90 days at Nouabalé-Ndoki National Park (Republic of Congo, 564 km from MDNP) (Morgan et al. 2006), and 88 days at Ebo Forest, Cameroon (250 km from MDNP) (Ndimbe et al. 2013). Such variation in nest decay rates despite proximity and similarity in habitat highlights the need for the acquisition of local nest decay rates. Although it is recommended whenever possible that decay rates be used from the same area and same time period as the survey data (Plumptre and Reynolds 1996; Ndimbe et al. 2013; Nzooh et al. 2015), we had no option but to use survey data collected from a different time than the present study. Our study aimed at assessing the nest decay rate for MDNP, which could be used to assess chimpanzee density in the area instead of using a non-local nest decay rate. Furthermore, testing other survey techniques such as mark nest count (Plumptre and Cox 2006; Ndimbe et al. 2013) and camera trap sampling (Howe et al. 2017; Agha et al. 2018), which do not depend on nest decay rates, are also important for achieving a reliable density estimate (Mathewson et al. 2008).

From our best fitted model (Model 2), there was no evidence of a significant direct correlation between the probability of nest decay and “topography”. The latter is a potentially overlooked variable which may influence tree cover and thus the availability of plants used for nesting and should be considered in future studies (Devos et al. 2008). Rainfall had an effect on the mean nest decay rate in the MDNP, increasing the probability of nest decay in the first month of the nest’s existence. The effects of rainfall on nest and dung decay rates have been reported in several studies (Barnes et al. 1997; Nchanji and Plumptre 2001; Walsh and White 2005; Ndimbe 2013). With older nests, the probability of decay increases dramatically with little or no effect of rainfall. As the monthly and annual precipitation fluctuates enormously between regions, it is important to take into account that this effect can vary strongly between sites, even when they are located in close proximity to one another (Walsh and White 2005).

Chimpanzee density and abundance in this study were similar to previous estimates in the MDNP (Maisels et al. 2009; Kamgang et al. 2018). These previous studies highlighted the magnitude of the variation in estimates based on the nest decay rate used, and how unreliable the density and abundance estimated might be when non-local nest decay rates are used to convert nest density into chimpanzee density. As many authors have reported, density estimates may be severely biased when using non-locally determined nest decay rates (Laing et al. 2003; Wich et al. 2004; Stokes et al. 2010). When using a mean nest decay rate of 120 days, our results are similar to those of the previous studies in MDNP. However, when comparing the extreme values (88 and 221 days) considered by Kamgang et al. (2018), the density and abundance of the chimpanzees were overestimated using 88 days, and underestimated using 221 days. Therefore, the assessment of locally determined nest decay rates is a crucial priority when applying the SCNC method for chimpanzee surveys.

It is also important to highlight that the heterogeneity in the nest decay rate may not only depend on the fluctuation of the climatic factors (such as rainfall), but other variables such as the nesting materials (tree species) and nest height (Laing et al. 2003; Wich et al. 2004; Mathewson et al. 2008). Also, factors like lignin content and the location of the nest in the tree may be considered as other factors which might influence nest decay rate and should be the focus of further investigations. In this study, we were only able to evaluate a small region compared to the extent of the entire national park.

The presence of the forest–savannah mosaic in MDNP increases the heterogeneity of both habitat characteristics and climatic conditions even more (Abwe et al. 2019) and, consequently, might also increase the geographical heterogeneity of the mean nest decay rate. At Ugalla, Tanzania, Stewart et al. (2011) and Hernandez-Aguilar et al. (2013) showed how environmental characteristics determined the nesting site choice, highlighting the effects of habitat heterogeneity on the nest decay rate variation. We recommend more surveys to be conducted in the forest–savannah mosaic area since few fresh nests were found in this habitat during our study. The use of the SCNC technique using locally acquired nest production and decay rates to estimate nest and animal densities is valuable in reducing biases in the long term. One strategy to avoid temporal heterogeneity biases of the nest decay rate could be to estimate correlations between decay rate and factors influencing this parameter such as habitat, rainfall, and nesting material. It might thus be possible to estimate the decay rate in a given time interval based on data obtained from these covariates during the period in which the survey was performed (Barnes et al. 1997). An effective solution to detect population trends and changes in population size is to carry out surveys repeatedly over long periods of time (Morgan et al. 2006). In addition, Mathewson et al. (2008) suggested enhancing the detection of short-term population changes by monitoring the main threats to the species at each site. Hence, an indirect estimation of the population variation could be obtained by measuring the magnitude of the fluctuations of these threats. We hope that by providing this local nest decay rate, our findings may help the MDNP management to more accurately assess chimpanzee population densities in order to shape their conservation strategies. Also, these results could alert other researchers to the importance of assessing site-specific nest decay rates over the distribution range of great ape species, which will be crucial to making accurate comparisons of population abundance across the landscape.

## Conclusions

In the present study, we estimated an in situ nest decay rate of 127 [95% (100–160)] days, for an estimated abundance of about 987 [95% (683–1427)] weaned chimpanzees in MDNP. With this figure, the MDNP should be considered as a site of exceptional priority for the conservation of Nigeria-Cameroon chimpanzee, given the large area of potential habitat and the long-term potential of the site (Morgan et al. 2011). Rainfall in a warm tropical region such as this plays an important role over the first period of a nest’s lifetime, significantly increasing the probability of nest decay. Other factors such as type of habitat or tree species should be considered in future studies, since they may also have effects on the mean nest decay rate (Laing et al. 2003; Walsh and White 2005; Mathewson et al. 2008). In order to improve the accuracy and precision of nest decay rates and density estimates, a larger area should be surveyed to obtain a more representative nest sample size for the whole national park and thus reduce the source of error based on spatial fluctuations of the nest decay rate. We recommend that researchers carry out repeated surveys over time in order to minimize the problem of temporal heterogeneity of this parameter as well as to detect short-term fluctuations of chimpanzee population densities (Maisels et al. 2009; Morgan et al. 2011). Finally, both the use of site-specific parameters as well as efficient threat mitigation are essential tasks for an effective management and conservation of great ape populations throughout their range.

## Electronic supplementary material

Below is the link to the electronic supplementary material.

Supplementary material 1 (PPTX 711 kb)

Supplementary material 2 (PPTX 818 kb)

Supplementary material 3 (PPTX 830 kb)

Supplementary material 4 (DOCX 13 kb)

Supplementary material 5 (DOCX 12 kb)
