# Supplementary figures and images for: Assessment of in situ nest decay rate for chimpanzees (Pan troglodytes ellioti Matschie, 1914) in Mbam-Djerem National Park, Cameroon: implications for long-term monitoring
Source: Primates. 2019 Oct 28;61(2):189–200. doi: 10.1007/s10329-019-00768-3 (PMC7080673; doi:10.1007/s10329-019-00768-3)

## Slide 1
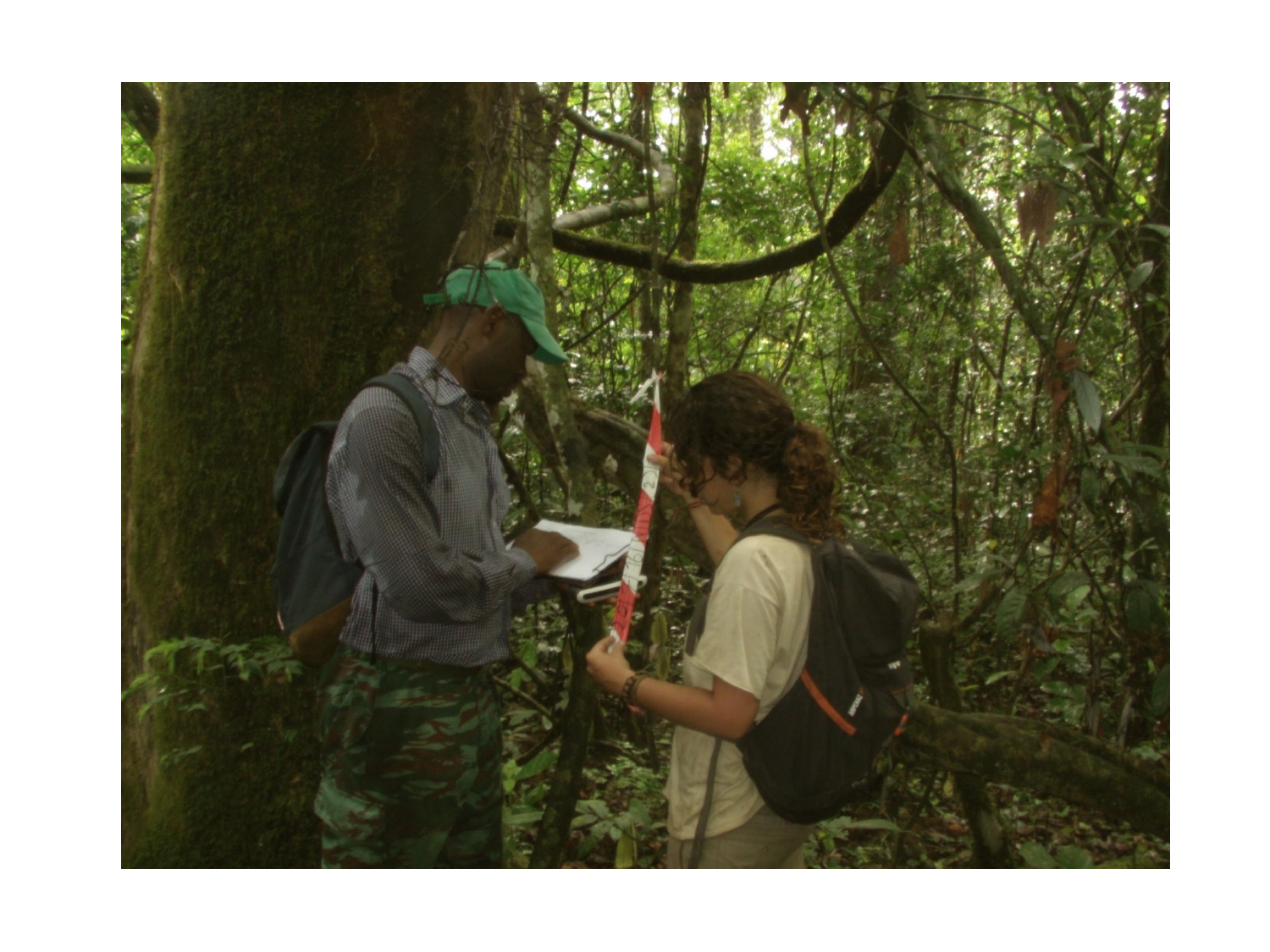

Supplement: Supplementary file 1 — Supplementary material 1 (PPTX 711 kb) [file 10329_2019_768_MOESM1_ESM.pptx]

## Slide 1
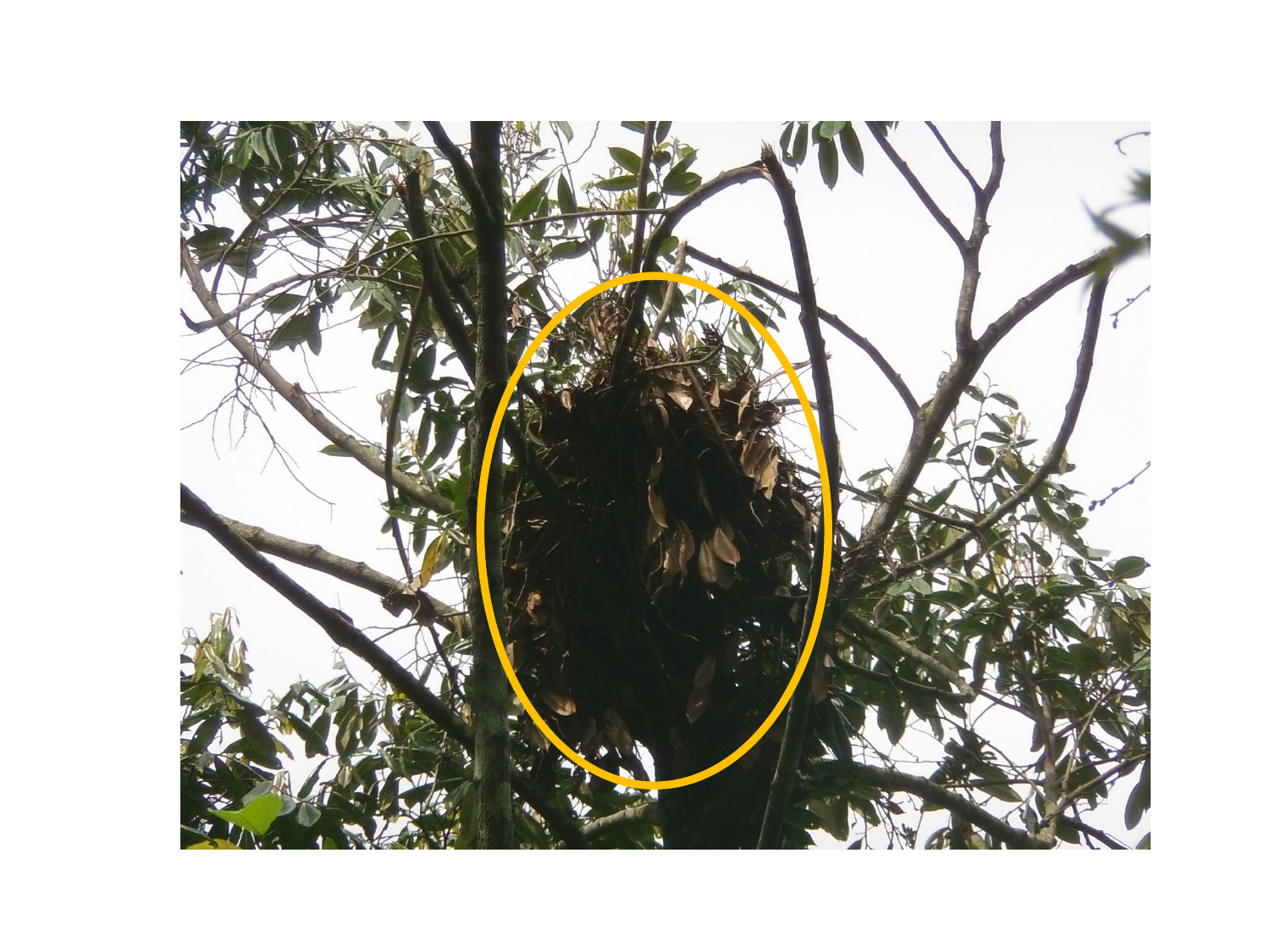

Supplement: Supplementary file 2 — Supplementary material 2 (PPTX 818 kb) [file 10329_2019_768_MOESM2_ESM.pptx]

## Slide 1
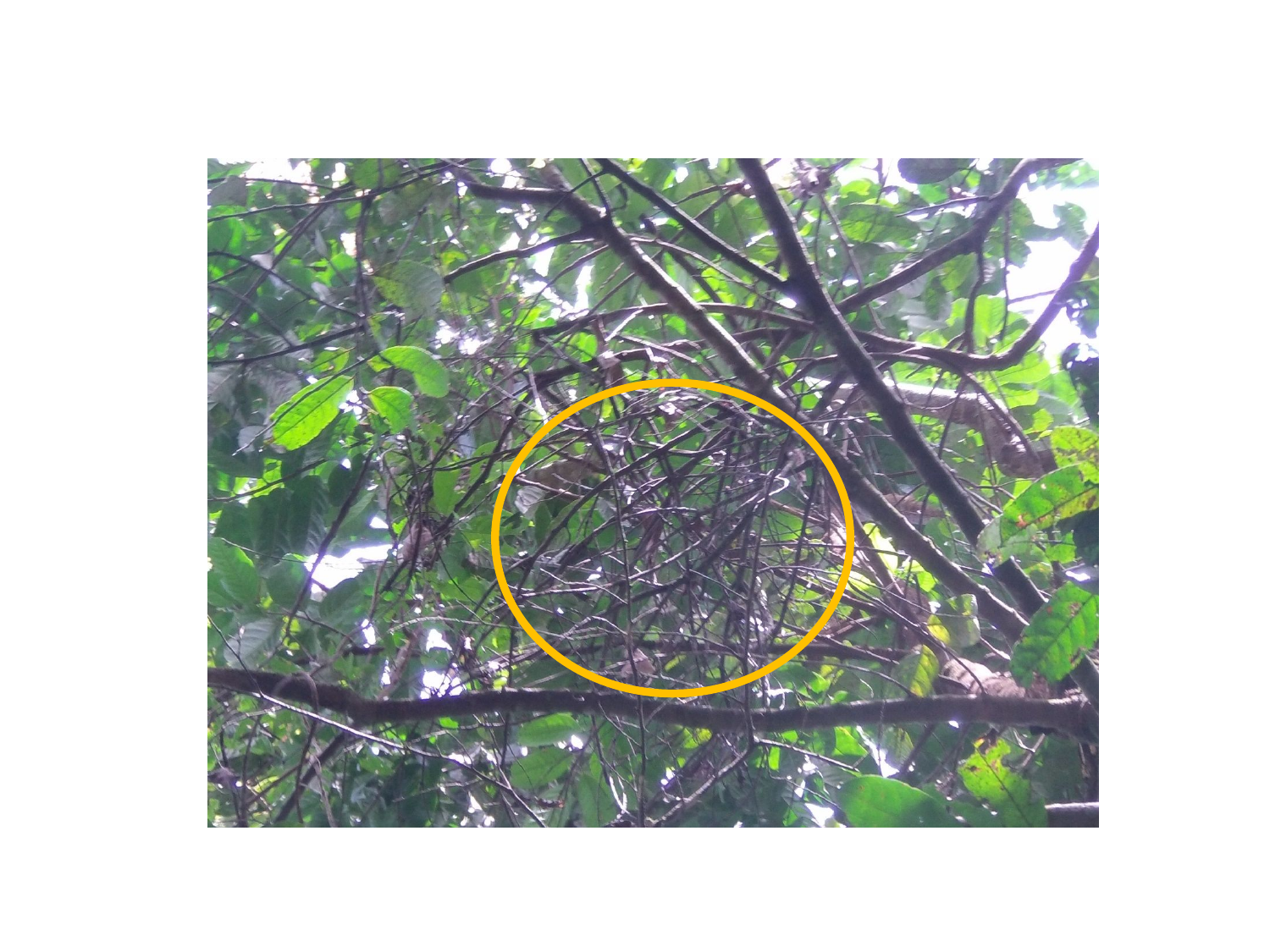

Supplement: Supplementary file 3 — Supplementary material 3 (PPTX 830 kb) [file 10329_2019_768_MOESM3_ESM.pptx]
