# Supplementary material for: Assessment of in situ nest decay rate for chimpanzees (Pan troglodytes ellioti Matschie, 1914) in Mbam-Djerem National Park, Cameroon: implications for long-term monitoring
Source: Primates. 2019 Oct 28;61(2):189–200. doi: 10.1007/s10329-019-00768-3 (PMC7080673; doi:10.1007/s10329-019-00768-3)
Supplement: Supplementary file 4 — Supplementary material 4 (DOCX 13 kb) [file 10329_2019_768_MOESM4_ESM.docx]

**Supplementary File 4:** Results obtained from fitting the three models in the second logistic regression analysis.

| **Models** | | **Residual deviance** | **Deviance change** | **df** | **df change** | **P** |
| --- | --- | --- | --- | --- | --- | --- |
| **Mode l** | Null  + Age  + Precipitation  +Topography  +Age*Topography  + Precipitation*Topography  + Age*Precipitation | 249.25  139.94  137.17  134.55  133.25  133.07  113.06 | 109.313  2.767  2.624  1.297  0.179  20.013 | 268  267  266  265  264  263  262 | 1  1  1  1  1  1 | <0.0001  0.0962  0.1052  0.2548  0.6725  <0.0001 |
| **Model 2** | Null  + (-1/Age)  + Precipitation  +Topography  + (-1/Age)*Topography  + Precipitation*Topography  +(-1/Age)* Precipitation | 249.25  122.00  118.27  115.18  112.08  112.07  110.64 | 127.251  3.730  3.092  3.101  0.007  1.428 | 268  267  266  265  264  263  262 | 1  1  1  1  1  1 | <0.0001  0.0534  0.0786  0.0782  0.9326  0.2321 |
| **Model 3** | Null  + (-1/√Age)  + Precipitation  +Topography  + (-1/√Age)*Topography  +Precipitation*Topography  + (-1/√Age)*Precipitation | 249.25  123.90  119.55  116.43  114.03  114.02  110.19 | 125.354  4.352  3.112  2.402  0.011  3.828 | 268  267  266  265  264  263  262 | 1  1  1  1  1  1 | <0.0001  0.0370  0.0777  0.1212  0.9165  0.0504 |

*“Absence/ Presence” was considered as the response variable and “Age”, “Precipitation”, “Topography” and their interactions as predictor variables.*
