# Supplementary material for: Assessment of in situ nest decay rate for chimpanzees (Pan troglodytes ellioti Matschie, 1914) in Mbam-Djerem National Park, Cameroon: implications for long-term monitoring
Source: Primates. 2019 Oct 28;61(2):189–200. doi: 10.1007/s10329-019-00768-3 (PMC7080673; doi:10.1007/s10329-019-00768-3)
Supplement: Supplementary file 5 — Supplementary material 5 (DOCX 12 kb) [file 10329_2019_768_MOESM5_ESM.docx]

**Supplementary File 5:** Results obtained from fitting the third logistic regression analysis in Model 2.

| **Models** | | **Residual deviance** | **Deviance change** | **df** | **df change** | **P** |
| --- | --- | --- | --- | --- | --- | --- |
| **Model 2** | Null  + (-1/Age)  +Precipitation  + Age*Precipitation | 367.35  123.25  118.77  117.77 | 244.101  4.477  1.730 | 308  307  306  305 | 1  1  1 | <0.0001  0.0344  0.1884 |

*“Absence/ Presence” was considered as the response variable and “Age”, “Precipitation” and their interactions as predictor variables.*
